# Psychosocial Factors Behind Deliberate Self-Poisoning in a Tertiary Care Hospital of Bangladesh: A Cross-Sectional Study

**DOI:** 10.7759/cureus.39893

**Published:** 2023-06-02

**Authors:** Binayak Bhattacharjee, Soumitra Roy, M. M. Jahangir Alam, R. K. S. Royle, Shrebash Paul, Md Sohidul Islam, Md. Shafiqul Bari, Fazle Rabbi Chowdhury

**Affiliations:** 1 Internal Medicine, Sylhet Shaheed Shamsuddin Ahmed Hospital, Sylhet, BGD; 2 Internal Medicine, Sylhet MAG Osmani Medical College Hospital, Sylhet, BGD; 3 Psychiatry, Sylhet MAG Osmani Medical College Hospital, Sylhet, BGD; 4 Infectious Disease and Tropical Medicine, Infectious Disease Hospital, Dhaka, BGD; 5 Internal Medicine, Bangabandhu Sheikh Mujib Medical University, Dhaka, BGD; 6 Internal Medicine, Dhaka Medical College Hospital, Dhaka, BGD; 7 Toxicology, Toxicology Society of Bangladesh, Dhaka, BGD

**Keywords:** deliberate self-poisoning, poisoning, psycho-social, depressive disorder, suicide

## Abstract

Introduction: Deliberate self-poisoning (DSP) is an important cause of hospital admissions and subsequent mortality. We conducted a cross-sectional observational study in a tertiary-level teaching hospital situated in the northeastern part of Bangladesh to analyze the psychosocial factors responsible for DSP.

Methods: This cross-sectional observational study was carried out among patients with DSP admitted to the medicine ward from January to December 2017, irrespective of gender, except for cases involving poisoning due to spoiled food, food contaminated by infectious organisms, poisoning by venomous animals, and street poisoning (commuter or travel-related poisoning). Consultant psychiatrist in accordance with the Diagnostic & Statistical Manual of Mental Disorder - IV (DSM-IV) confirmed psychiatric disorders. Data were analyzed by SPSS (Statistical Package for social sciences) version 16.0 (IBM Corp., Armonk, NY).

Results: Total 100 patients were enrolled. Among them, 43% were male and 57% were female. The majority (85%) of the patients were young, aged below 30 years. The mean age of male patients was 26.2 years and that of females was 21.69 years. Most of the DSP patients were from the lower economic class (59%). The population sample was remarkable for students (Prevalence 37%). The highest percentage of patients (33%) had their educational status at the secondary level. The common reasons for DSP were a family problem in 31% patients, quarrel with boy/girlfriend in 20%, quarrel with a spouse in 13%, quarrel with parents or other family member in 7%, failure in examination in 6%, poverty in 3%, and unemployment in 3%. Prescription medication was the most common poison material (38%), followed by insecticides (36%), household cleaners (17%), and rodenticides (8%). Seven (7%) patients reported previous deliberate self-harm events and co-morbid psychiatric disorder was present in 30% patients among them major depressive disorder was found in 60%, and schizophrenia in 23.3% cases.

Conclusion: DSP remains a problem mainly for the young with gender ratio-favoring females. The majority of DSPs were educated up to secondary level, unmarried, residents of rural areas, student, and belonged to the lower class. Familial disharmony and quarrel with spouse or friends were the common reason behind DSP. Prescription medication and insecticides were commonly used for DSP. Psychiatric disorders, primarily depressive disorder, and schizophrenia were common in cases of DSP.

## Introduction

Deliberate self-poisoning (DSP) is an important health problem throughout the world especially in developing countries [1]. It is a major cause of more than 500,000 deaths per year in the Asia Pacific region [2]. It is an important health issue in the context of adolescents and younger populations. Young, married females constitute the majority of persons attempting deliberate self-harm [3]. The commonest compound used for DSP varies at different places. In developing countries, benzodiazepines and organophosphorus (OP) pesticides are used commonly in urban and rural areas, respectively [1,3]. Persons attempting DSP may have various intentions, of which, using it as a means of communication, threatening and suicidal are the most important [4]. The majority of the persons attempting DSP do not want to commit suicide [5]. They do so as they are depressed, have anger, jealousy, or desire for attention [4]. OP compounds are generally used for DSP with suicidal intentions because of their well-known fatality index [1].

Mental disorders and previous attempts at DSP are associated with poor outcomes like death. In Bangladesh, pesticide poisoning accounted for 39.1% of total poisoning cases and OP compound constitutes 89.8% while pesticide poisoning causes approximately 0.7 deaths per 100,000 population [6]. The poisoning agents in Bangladesh are different because of the social structure, economic status, educational level, awareness of the people, and availability of drugs [7]. The important reasons for suicidal poisoning in Bangladesh are lack of education, frustration, familial disharmony, failure of love affairs, failure in the examination, and the availability of the poison [7,8]. The socio-economic condition of people in the developing world has led to a higher suicidal rate as compared to the Western world. The pattern of poisoning changes over time and varies from country to country and even within regions of countries [9,10]. This study tried to identify the possible psychosocial factors responsible for DSP among patients admitted to a tertiary hospital in Bangladesh.

## Materials and methods

This cross-sectional observational study was carried out in the Department of Medicine, Sylhet MAG Osmani Medical College Hospital, Sylhet, Bangladesh from January to December 2017. Sylhet M.A.G. Osmani Medical College Hospital is the largest medical college hospital in the northeastern part of the country. It has more than 1,000-bed capacity with a bed occupancy rate of 162%. The inclusion criteria of our study were all patients with DSP of 18 years and above irrespective of gender and all who gave consent; Exclusion criteria were accidental poisoning (poisoning due to spoiled food, food contaminated by infectious organisms), poisoning by venomous animals, and street poisoning. Socio-economic status has been categorized as “Higher class” having a monthly family income of more than Tk. 20,000.00, “Middle class” having a monthly family income of Tk. 10,000.00 to 20,000.00 and “Lower class” having monthly family income of less than Tk. 10,000.00. A psychiatric assessment was done after the recovery. A psychiatric interview was to elicit the symptoms, history, and background information from both the patient and informant, and a mental state examination was done by the psychiatric consultant. The patient and informant were asked to describe the course of the psychiatric symptoms if any, family history, personal history, previous medical and psychiatric history along with previous personality history. Psychological assessment of DSP patients was categorized using the Diagnostic & Statistical Manual of Mental Disorder- IV (DSM-IV)-TR. The sample size was calculated using Cochran's formula considering a 5% level of significance, a 5% precision level (marginal error), and estimating the prevalence of poisoning as 7.1% [6]. Purposive random sampling was employed as a sampling technique in this study. Every day at least three new patients with DSP were randomly approached and consent was recorded. Data were processed manually and analyzed with the help of SPSS (Statistical Package for social sciences) version 16.0 (IBM Corp., Armonk, NY). Quantitative data were expressed as mean and standard deviation, and comparisons were done by the “Z” test. Discrete and categorical variables were expressed as frequency and percentage; the comparison was carried out by Chi-square (χ2) Test. A probability value (p) of less than 0.05 was considered to indicate statistical significance.

## Results

A total of 100 patients were included in this study. Out of them, 57% were female. The age of the patients ranged from 18 to 65 years. The majority (85%) of the patients were young, aged below 30 years, 10% of cases between 31 and 40 years and rest were above 41 years. Among the total patients, 67% were unmarried, 86% patients were Muslim, 73% patients were from a nuclear family, 59% were patients from rural, 30% were patients from urban and 11% patients were from sub-urban society. Most of the DSP patients (59%) were from the lower socio-economic class, followed by the middle class (40%). About one-third of the DSP patients were students (37%). Out of the remaining two-thirds, 16% were homemakers, 12% were unemployed, 10% were day laborers and 09% were farmers. Most of the DSP patients in this study were educated up to various levels and 12% were illiterate (Table 1).

**Table 1:** Socio-demographic characteristics of DSP patients (n=100)

Socio-demographic Variables	Percentage
Age distribution with Mean age & Range(Years)	
All , 23.6 & (14-65)	100 (n=100)
Male , 26.2 & (16-65)	43 (n=43)
Female , 21.7 & (14-34)	57 (n=57)
Age group (years)	
<20	46
21-30	39
31-40	10
41-50	03
51-60	01
>60	01
Gender	
Male	43
Female	57
Education	
Illiterate	12
Primary	26
Secondary	33
Higher secondary	19
Graduate	09
Post graduate	01
Marital status	
Married	33
Unmarried	67
Occupation	
Students	37
Housewife	16
Unemployed	12
Others	12
Day laborer	10
Farmer	09
Service	02
Business	02
Social background	
Rural	59
Urban	30
Semi urban	11
Religion	
Islam	86
Hinduism	14
Family type	
Nuclear	73
Joint family	27

Interpersonal relationship problem (quarrel with a boy/girlfriend or with spouse) was the most common (33%) reason followed by familial disharmony (31%). Other reasons behind DSP were quarrels with parents or other family members (7%), failure in examination (6%), poverty (3%), unemployment (3%) and others (17%) (Table 2). Age and occupation had significant relation with the cause of DSP (p<0.05). In most of the patients below 30 years of age, the cause for DSP is family problems (61.2%). Familial disharmony and quarrel with boy/girlfriend (27% in each case) was the most common reason for DSP amongst students. DSP among homemakers is more commonly due to quarrels with spouses (43.8%). Prescription medication ingestion was the most common method (38%), followed by ingestion of insecticides (36%), household cleaners (17%), rodenticides (8%) and others (1%) (Table 2).

**Table 2:** Clinical characteristics of DSP patients (n=100)

Clinical Condition	Percentage
Reason behind DSP	
Familial disharmony	31
Quarrel with boy/girlfriend	20
Quarrel with spouse	13
Quarrel with parents or family members	07
Failure in examination	06
Poverty	03
Unemployment	03
Others	17
Types of poison materials used	
Prescription medication ingestion	38
Pesticides, insecticides	36
Household cleaner	17
Rodenticide	08
Others	01
Co-morbid specific psychiatric disorders	
Major depressive disorder	18
Schizophrenia	07
Bipolar affective disorder	02
Substance use disorder	02
Post-traumatic stress disorder	01
No psychiatric disorder	70

Education, occupation, economic and social background has more influence on the type of poison material used for DSP. Illiterate and patients educated up to a primary and secondary level more commonly used insecticides (illiterate - 91.7%, primary - 42.3%, secondary - 33.3%) whereas patients with a higher level of education used drugs more commonly (higher secondary - 73.7%, graduate - 66.7%). In terms of occupation, there is some variability in the types of poisons used by different occupations. Most of the students and homemakers used drugs (students - 54.1%, homemakers - 62.5%) and most farmers, day laborers and unemployed people used insecticides for DSP (farmers - 66.7%, day laborers - 80%, and unemployed - 66.7%) (Table 3).

**Table 3:** Relation of occupation and type of poisons used

		Type of poisons					Total	p- value
		Insecticides	Prescribed drug ingestion	Household cleaner	Rodenticide	Others		
Occupation	Service	0	0	2	0	0	2	.000
	Business	0	2	0	0	0	2	
	Farmer	6	2	0	1	0	9	
	Student	4	20	10	3	0	37	
	Homemaker	5	10	0	1	0	16	
	Unemployed	8	1	3	0	0	12	
	Day laborer	8	1	1	0	0	10	
	Others	5	2	1	3	1	12	
Total		36	38	17	8	1	100	

Patients belonging to lower socioeconomic classes more commonly used insecticides (52.5%) and middle-class patients used drugs (70%) for DSP. Rural people more commonly used insecticides (54.2%) and people residing in the urban and suburban areas used drugs more commonly (urban - 70%, suburban - 63.6%). The source of poison materials was buying from the local shop in 37% of cases, household materials in 37%, and prescribed medications in 16% of patients.

Seven (7%) patients reported previous attempts of deliberate self-harm. A co-morbid psychiatric disorder was present in 30% of cases. Among them, major depressive disorder (MDD) was the most common psychiatric disorder (60%), followed by schizophrenia (23.3%), bipolar affective disorder (6.7%), substance use disorder (6.7%) and post-traumatic stress disorder (3.3%) cases. The source of poison and different type of psychiatric disorders has a significant relationship with each other (p-value=0.04). In 72.2% of cases of patients with MDD bought poison from shops whereas in 85.7% cases of patients with schizophrenia got poisoned by household materials (Table 4).

**Table 4:** Relation of source of poison and type of psychiatric disorder Chi-square and fishers exact test was used to explore the level of significance

		Type of psychiatric disorder						Total	p- value
		No psychiatric disorder	MDD	Schizophrenia	Bipolar affective disorder	PTSD	substance abuse disorder with impulsivity		
Source of poison	Household materials	26	3	6	1	1	0	37	0.04
	Shop	32	13	0	0	0	2	47	
	Prescribed drugs	12	2	1	1	0	0	16	
Total		70	18	7	2	1	2	100	

## Discussion

The male-to-female ratio in this study was 1:1.35. DSP is an indirect indicator of psychological distress, and the finding on male/female differences suggested that females probably faced more psychological distress than males. The fact also indicates that females in Bangladesh face different difficulties such as domestic violence and abuse. In previous studies done in Bangladesh, males were found to be involved more than females. Those studies included street or commuter poisoning in the data which probably drew the difference. Males are more mobile and thus can easily become victims of street poisoning [11-16].

In this study, the mean age of the female patients was significantly lower than that of male patients (p<0.01). The result is concordance with the study of Srivastava et al. [17] and Akbaba et al. [18]. Amin et al. showed that the mean age (± SD, years) of the poisoning patients was 26±13 years with a wide variation in the range probably due to the inclusion of accidental poisoning and snakebite in that study [11]. In all of these studies, the mean age for DSP patients was found to be under 30 years. This result is similar to a baseline survey on poisoning cases done by Faiz, which showed a majority of DSP patients belonged to 13-20 years [12]. Below 30 years age group was also found to be more vulnerable to poisoning in a study done by Chowdhury et al. [13]. However, another study done by Howlader et al. [14] showed that the majority of poisoning cases belonged to 36-45 years (40%), which was due to involvement of street poisoning (commuter/travel related) and again, this age group is more active and mobile than others and more vulnerable to street poisoning.

Teenagers are more vulnerable to DSP because they are more prone to be emotionally impulsive. They begin to develop their own existence and want to come out of the guardianship of parents or seniors and there may be a virtual conflict between two generations. In this age group, attraction to the opposite gender is at its extreme. So, there is every possibility of having a boy/girlfriend, and quarrels between them or breaking up makes them vulnerable to DSP [5,11].

Most of the patients belong to a low socio-economic group which is similar to other studies done in Bangladesh [7,8,11,13]. People from lower socio-economic groups are exposed to social and economic deprivation leading to a stressful life. Furthermore, people from the upper socio-economic class are less willing to get admitted to government hospitals. This is probably the reason behind having less patients of the upper socio-economic class in this study.

Patients belonging to lower socioeconomic class (52.5%) more commonly used insecticides and middle-class patients (70%) used the drugs as an offending agent. Agriculture is the main means of living for lower economic class people and they have easy access to pesticides. This study showed that 73% of the DSP patients were from the nuclear family and 27% of patients were from the joint family. This result is similar to the study of Srivastava et al. and Narang et al. [17,19]. People living in the nuclear family become self-centered. Usually, they have less chance to get knowledge of customs and religion from elders, i.e., grandfather or grandmother or other family members than parents. This may be the cause of the difference found in this study among joint and nuclear families. Emotional support in a joint family can also be a protective factor against DSP.

In this study, one-third (37%) cases were students, followed by housewives (16%), unemployed (12%), and others, e.g., day laborers, farmers, business, and service. Other studies conducted in different countries also found the same [16,20,21]. In our study, familial disharmony and interpersonal relationship problems (quarrel with boy/girlfriend) were the most common reasons behind DSP in students. Quarrel with spouse was the major reason behind DSP amongst homemakers. The majority of homemakers involved in DSP faced domestic violence and abuse. The most common method of DSP amongst students and homemakers was the use of drugs and most of the farmers, day laborers, and unemployed person used insecticides. This may be due to the availability of the substance to a particular group of people.

A low educational status was found in one-third of the cases in this study. A similar result was reported by some other studies [14,20,22]. However, this result differs from the study done by Srivastava et al. [17] in India which found that more than half of the patients were illiterate. DSP patients educated up to primary and secondary levels more commonly used pesticides, whereas patients with higher levels of education commonly used drugs. This could be explained by the lack of knowledge of less educated person regarding the lethality of pesticides or there may be a strong intention for suicide. In this study, most of the DSP patients (59%) came from rural areas, compared to urban (30%) and sub-urban (11%). These findings were in accordance with the findings of Amin et al. in Dhaka which showed that 60% of poisoning patients live in rural areas [11]. Andrus et al. found a difference in the USA where 19.8% of suicide attemptees lived in urban, 40.3% in rural and 39.8% in suburban areas [23]. However, some other studies showed that poisoning patients were mostly city or urban dwellers [15,20]. Rural people more commonly used insecticides (54.2%) and people residing in urban and suburban areas used drugs more commonly for DSP (urban - 70%, suburban - 63.6%). In rural areas, agents like organophosphate, carbamate, and others are easily available and people living in rural areas are more familiar with these substances. Moreover, they are less aware of the deleterious effects of poisoning. People residing in urban or suburban areas can easily get drugs either from home or buy from a pharmacy. In terms of religion, the Muslim population was most prevalent (86%) among DSPs as Bangladesh is a Muslim-majority country (91.04%) [24]. Although suicide is forbidden in every religion including Islam, however, those who commit DSP are not always influenced by religion. That is why one of the objectives of our study was to explore background psychiatric conditions that dragged them to DSP, and we found that 30% have a psychiatric illness.

The common reasons behind DSP were found to be family disharmony, interpersonal relationship conflict, failure in examination, poverty, and unemployment. The finding is similar to other studies done in Indonesia and Ethiopia [25,26]. In terms of poisoning agents, the findings are similar to previous baseline studies done in Bangladesh [11,12], where prescription medication mainly sedatives were found to be the most common agent, followed by pesticides, household corrosives, and others. The findings were also similar in Japan (55.5% prescription medication) and Iran (60.8% prescription medication) [15,27]. However, Chowdhury et al. [13] reported a different scenario from the southern part of Bangladesh. The most commonly found toxic agents were organo-phosphate compound (27.6%) followed by unknown substance (16%), copper-sulfate (14%), and sedatives (13.4%) [13]. The reason for such a high ratio of prescription medication as a method of DSP is probably due to the extensive prescribing of these medications and their easy availability at home. Moreover, most medication can be bought without a prescription in Bangladesh.

Seven (7%) of the participants had a previous history of DSP and five (5%) of the respondents had a history of a previous established psychiatric disorder. In Romania, Sorodoc et al. found that a total of 77.8% of patients were the first suicide attempter, 11.7% of patients had a history of two suicide attempts and 10.5% had records of more than two attempts [28]. Yamada et al. found that attempted suicide was the first time in 56.4%, second time in 22.5%, and third times or above in 21.1% of the cases [27]. Sorodoc et al. reported a history of psychiatric illness in 13.9% of the cases [28].

This study identified psychiatric disorders in 30% of DSP patients. A systematic review and meta-analysis by Knipe et al. also found a low prevalence of psychiatric disorders in suicidal tendency in low- and middle-income countries (an average of 45% in non-fatal suicidal tendency and 55% in those who died by suicide) compared to high-income countries (80%-90%) [29]. The common conditions we found in our study were MDD, schizophrenia, bipolar affective disorder, substance use disorder, and post-traumatic stress disorder. The findings were also supported by other studies [19,25,27] reported from different parts of the world. Community engagement and intervention (CEI) activities seriously lack in these psychiatric disorder issues. More CEI activities and addressing mental health issues could reduce the events.

Our study has some limitations. Although it is an observational study, the sample size is small. We could not discuss the difference between suicide attempts versus self-injurious behaviors like cutting. The conclusion was based on the limited evidence that we got from admitted patients with DSP. Therefore, it is probably not the true reflection of the community.

## Conclusions

DSP with various agents including pesticides and sedatives is one of the common medical emergencies in Bangladesh. We often overlook the background psychiatric conditions of the patients, which might drag one to this horrible situation. Psychiatric evaluation should be done in every case of DSP to prevent recurrence. Diagnosing the actual cause and addressing the conditions could save lives.

## References

[1] Eddleston M (2000). Patterns and problems of deliberate self-poisoning in the developing world. QJM An Int J Med.

[2] Eddleston M, Phillips MR (2004). Self poisoning with pesticides. BMJ.

[3] Khan MM, Reza H (1998). Gender differences in nonfatal suicidal behavior in Pakistan: significance of sociocultural factors. Suicide Life Threat Behav.

[4] Khurram M, Mahmood N (2008). Deliberate self-poisoning: experience at a medical unit. J Pak Med Assoc.

[5] Eddleston M, Sheriff MH, Hawton K (1998). Deliberate self harm in Sri Lanka: an overlooked tragedy in the developing world. BMJ.

[6] Dewan G (2014). Analysis of recent situation of pesticide poisoning in Bangladesh: is there a proper estimate?. Asia Pacific J Med Toxicol.

[7] Howlader MAR, Sardar MH, Amin MR, Morshed MG, Islam MS, Uddin MZ, Azhar MA (2010). Clinico-epidemiological pattern of poisoning in a tertiary level hospital. J Dhaka Med Coll.

[8] Hossain R, Amin R, Riyadh Hossain A, Kahhar A, Rabbi Chowdhury F (2016). Clinico-epidemiological study of poisoning in a tertiary care hospital in Bangladesh. J Emerg Pract Trauma.

[9] Lee HL, Lin HJ, Yeh ST, Chi CH, Guo HR (2008). Presentations of patients of poisoning and predictors of poisoning-related fatality: findings from a hospital-based prospective study. BMC Public Health.

[10] Hendrix L, Verelst S, Desruelles D, Gillet JB (2013). Deliberate self-poisoning: characteristics of patients and impact on the emergency department of a large university hospital. Emerg Med J.

[11] Amin MR, Awwal A, Sattar MA (2009). Pilot survey on cases of poisoning and its outcome in different category of hospitals in Bangladesh. J Med.

[12] Amin MR (2017). Baseline survey on cases of poisoning and its outcome in Bangladesh. Open Access J Toxicol.

[13] Chowdhury FR, Rahman AU, Mohammed FR, Chowdhury A, Ahasan HA, Bakar MA (2011). Acute poisoning in southern part of Bangladesh--the case load is decreasing. Bangladesh Med Res Counc Bull.

[14] Howlader MAR, Hossain MZ, Morshed MG, Begum H, Sardar MH, Uddin MZ, Azad KAK (2011). Changing trends of poisoning in Bangladesh. J Dhaka Med Coll.

[15] Islambulchilar M, Islambulchilar Z, Kargar-Maher MH (2009). Acute adult poisoning cases admitted to a university hospital in Tabriz, Iran. Hum Exp Toxicol.

[16] Khadka SB, Khadka SB (2005). A study of poisoning cases in emergency Kathmandu Medical College Teaching Hospital. Kathmandu Univ Med J (KUMJ).

[17] Srivastava MK, Sahoo RN, Ghotekar LH, Dutta S, Danabalan M, Dutta TK, Das AK (2004). Risk factors associated with attempted suicide: a case control study. Indian J Psychiatry.

[18] Akbaba M, Nazlican E, Demirhindi H, Sütoluk Z, Gökel Y (2007). Etiological and demographical characteristics of acute adult poisoning in Adana, Turkey. Hum Exp Toxicol.

[19] Narang RL, Mishra BP, Nitesh M (2000). Attempted suicide in ludhiana. Indian J Psychiatry.

[20] Akkose S, Bulut M, Armagan E, Cebicci H, Fedakar R (2005). Acute poisoning in adults in the years 1996-2001 treated in the Uludag University Hospital, Marmara Region, Turkey. Clin Toxicol (Phila).

[21] Molero P, Grunebaum MF, Galfalvy HC (2014). Past suicide attempts in depressed inpatients: clinical versus research assessment. Arch Suicide Res.

[22] Fathelrahman AI, Ab Rahman AF, Zain ZM, Tengku MA (2006). Factors associated with adult poisoning in northern Malaysia: a case-control study. Hum Exp Toxicol.

[23] Andrus JK, Fleming DW, Heumann MA, Wassell JT, Hopkins DD, Gordon J (1991). Surveillance of attempted suicide among adolescents in Oregon, 1988. Am J Public Health.

[24] (2023). Census 2022: number of muslims increased in the country. https://www.dhakatribune.com/bangladesh/2022/07/27/census-2022-number-of-muslims-increased-in-the-country.

[25] Kurihara T, Kato M, Reverger R, Tirta IG (2009). Risk factors for suicide in Bali: a psychological autopsy study. BMC Public Health.

[26] Desalew M, Aklilu A, Amanuel A, Addisu M, Ethiopia T (2011). Pattern of acute adult poisoning at Tikur Anbessa specialized teaching hospital, a retrospective study, Ethiopia. Hum Exp Toxicol.

[27] Yamada T, Kawanishi C, Hasegawa H (2007). Psychiatric assessment of suicide attempters in Japan: a pilot study at a critical emergency unit in an urban area.. BMC Psychiatry.

[28] Sorodoc Sorodoc, Victorita Jaba, Irina M Lionte, Catalina Mungiu, Ostin C Sorodoc, Laurentiu Laurentiu (2011). Epidemiology of acute drug poisoning in a tertiary center from Iasi County, Romania.. http://www.ncbi.nlm.nih.gov/pubmed/20630913.

[29] Knipe D, Williams AJ, Hannam-Swain S (2019). Psychiatric morbidity and suicidal behaviour in low- and middle-income countries: a systematic review and meta-analysis. PLoS Med.

